# Synthesis and Structural Evolution of AgCuCoNiFe High-Entropy Alloy via a Precipitation–Reduction Route

**DOI:** 10.3390/ma19091743

**Published:** 2026-04-24

**Authors:** Tomasz Michałek, Katarzyna Skibińska, Konrad Wojtaszek, Marek Wojnicki, Piotr Żabiński

**Affiliations:** Faculty of Non-Ferrous Metals, AGH University of Krakow, Al. Adama Mickiewicza 30, 30-059 Kraków, Poland; tomaszm@agh.edu.pl (T.M.); kwojtasz@agh.edu.pl (K.W.); marekw@agh.edu.pl (M.W.); zabinski@agh.edu.pl (P.Ż.)

**Keywords:** high-entropy alloys, AgCuCoNiFe, precipitation-reduction, co-precipitation, surface segregation, structural evolution, XRD, wettability

## Abstract

High-entropy alloys (HEAs) are typically produced using high-temperature metallurgical routes; however, alternative synthesis approaches based on wet-chemical processing remain relatively unexplored. In this study, a compositionally complex two-phase AgCuCoNiFe high-entropy alloy was synthesized using a precipitation–reduction strategy involving co-precipitation of mixed metal carbonates followed by thermal reduction in a reducing atmosphere. The objective of the work was to evaluate the feasibility of this hydrometallurgical route for preparing compositionally complex alloys and to investigate the structural evolution of the material as a function of reduction time. Quantitative MP-AES analysis confirmed efficient co-precipitation of all five elements, enabling the preparation of a precursor with near-equimolar metal composition. Structural characterization using SEM, EDS, and XRD revealed the presence of surface compositional heterogeneity in the as-reduced state, characterized by Ag-enriched domains. After controlled surface abrasion, the internal material exhibited significantly more uniform elemental distribution, although the obtained composition was not equimolar. X-ray diffraction patterns showed a transition from multiple sharp reflections at the surface to broadened peaks in the bulk, consistent with enhanced alloying within the bulk compared to the surface, while still revealing a two-phase character. Microhardness measurements indicated moderate hardness with mean values in the range of 187–221 HV with no significant dependence on reduction time, while wettability analysis revealed moderately hydrophilic behavior with contact angles in the range of approximately 75–83°. The results suggest that precipitation–reduction can be a viable alternative route for the synthesis of multicomponent HEAs, enabling the formation of chemically mixed alloy structures without the use of conventional melting-based processing. However, the obtained alloy exhibits incomplete chemical homogeneity, indicating that further optimization of the synthesis conditions is required to achieve a fully uniform composition.

## 1. Introduction

High entropy alloys (HEAs) are alloys of 5 or more elements with the concentration of each element in the range between 5 and 35 at.% [1]. This results in entropy of mixing values higher than 1.5R [2]. First works describing multicomponent [3] and high-entropy alloys [4,5] were published in 2004. Since then, the topic has attracted increasing interest. According to the Scopus database, approximately 1200 publications connected to the keyword ‘high-entropy alloys’ in 2026. Their popularity relates to their unique properties due to the observed four ‘core effects’: thermodynamic high entropy effect, structural lattice distortion effects, kinetic sluggish diffusion effect, and the “cocktail” effect [6]. Additionally, fabricated high-entropy alloys can then be modified using various methods, e.g., anodization, chronoamperometric oxidation, or oxidation in a furnace [7].

Atomic size differences and thermodynamic parameters such as the enthalpy and entropy of mixing, as well as the melting temperature of the constituent elements, must be considered when designing the composition of HEAs because they strongly influence phase stability and solid-solution formation [8]. As a consequence, the properties of high-entropy alloys are strongly governed by their phase constitution and resulting microstructure. Depending on alloy composition and processing conditions, HEAs may form single-phase solid solutions with face-centered cubic (FCC) or body-centered cubic (BCC) crystal structures, but multiphase microstructures are also frequently observed [9]. In many cases, elemental interactions and thermodynamic incompatibilities lead to phase separation or compositional partitioning within the alloy, resulting in the formation of double-phase systems [10]. These microstructures may consist of phases with different chemical compositions resulting from elemental partitioning, where regions enriched in specific elements coexist with a matrix containing the remaining components [11]. Such phase-separated HEAs can significantly influence the mechanical behavior of the material, as differences in strength and deformation characteristics between the constituent phases lead to strain partitioning and heterodeformation-induced strengthening during plastic deformation [12]. Therefore, understanding the relationship between alloy composition, thermodynamic parameters, and the resulting microstructure is essential for the rational design and optimization of high-entropy alloys. The application of nano-additives in the lubrication of mechanical transmissions has demonstrated that precise control over material and fluid composition can significantly enhance system efficiency. In this context, understanding the phase constitution of high-entropy alloys provides a foundation for designing surfaces with improved functional and tribological properties [13].

There are three main approaches to design HEAs [14]. The first one, Ming-Hung Tsai connected with the focus on a particular application, e.g., Refractory HEAs [15] or High-Entropy Superalloys (HESA) [16]. The second is based on modifying the composition of a conventional alloy by replacing the solvent or the solutes with additional elements [17]. The last one is about incorporating the second phase directly in the cast, or as precipitations in a matrix after heat treatment. This approach is used to enhance the mechanical properties of HEAs [18,19]. The design of HEAs is usually made with the use of the thermodynamic theoretical calculations [20], the CALculation of PHAse Diagrams (CALPHAD) method [21,22], the first-principles approach [23,24], and machine-learning algorithms [25,26,27].

There are many methods of high-entropy alloy fabrication, e.g., Laser Cladding [28,29,30], Mechanical Alloying (MA) and Spark Plasma Sintering (SPS) [31,32,33,34], Electrodeposition [35,36,37] and Additive Manufacturing (AM) [38,39,40]. Their choice depends on the used components, the required final form of the material, and the desired phase composition. An alternative approach to alloy synthesis involves a precipitation-reduction pathway conducted entirely through wet-chemical processing. This approach was previously applied by the authors in the synthesis of AgCu alloy powders [41]. In this strategy, metal ions are combined in solution, co-precipitated as mixed solid precursors, and subsequently converted into metallic alloys via thermal treatment in a reducing atmosphere. Although the final composition may be influenced by precipitation equilibria and incomplete conversion of ions from solution, the overall alloy stoichiometry can be tailored by adjusting the initial solution chemistry and processing parameters. The key advantage of this method lies in the intimate mixing of elements at the ionic level prior to alloy formation, which may facilitate improved elemental mixing compared to conventional melting-based routes. Moreover, the relatively low processing temperatures and powder-based character of the technique make it particularly attractive for the preparation of compositionally complex systems. Extending this precipitation-reduction concept from binary alloys to multicomponent systems offers a promising pathway for the synthesis of high-entropy alloy powders, where uniform elemental distribution and controlled microstructure are essential for achieving desirable mechanical and functional properties.

In the present work, a multicomponent AgCuCoNiFe HEA was synthesized using an innovative precipitation-reduction strategy based on co-precipitation of metal carbonates followed by controlled thermal treatment. Unlike conventional HEA synthesis routes, which typically rely on high-temperature melting processes, the proposed approach proceeds entirely in the solid state and bypasses the liquid metal phase. This wet-chemical pathway enables the preparation of compositionally complex alloys starting from well-mixed precursor mixture while avoiding the limitations associated with melting, such as high energy consumption. The study aimed to evaluate the feasibility of this approach for preparing a compositionally complex alloy and to examine the structural evolution as a function of reduction time. The results demonstrate that the applied processing route leads to the formation of a compositionally complex, two-phase HEA system with partial chemical mixing. The key significance of this work lies in the introduction and validation of a novel precipitation–reduction synthesis route for high-entropy alloys, which differs fundamentally from conventional melting-based techniques. This approach provides a new pathway for producing compositionally complex HEAs with lower processing temperatures, and reduced energy requirements, thereby offering a promising alternative for scalable and efficient fabrication of advanced alloy systems. These findings confirm that the precipitation-reduction pathway can be successfully extended from binary systems to multicomponent high-entropy alloys.

## 2. Materials and Methods

### 2.1. Synthesis of HEA

Copper(II) sulfate pentahydrate (6.4 g, analytical grade, Chempur, Piekary Śląskie, Poland), Nickel(II) sulfate hexahydrate (6.75 g, 99% purity, Acros Organics, Geel, Belgium), Cobalt(II) sulfate heptahydrate (7.21 g, analytical grade, Chempur, Piekary Śląskie, Poland), Silver(I) sulfate (4.0 g, analytical grade, POCH, Gliwice, Poland), and Iron(III) sulfate x-hydrate (6.1 g, analytical grade, POCH, Gliwice, Poland) were used as precursors. As iron(III) sulfate does not possess a strictly defined and constant hydration state under ambient conditions, the actual iron content in the supplied hydrate was determined analytically prior to solution preparation using an AGILENT 4200 MP-AES microwave plasma-atomic emission spectrometer (Agilent, Santa Clara, CA, USA). Before sample analysis, the MP-AES instrument was calibrated for all investigated metals, including Ag, Cu, Co, Ni, and Fe. Calibration solutions in the range of 0–20 ppm were prepared by diluting certified 1000 ppm single-element standards (PlasmaCAL, Analytika Ltd., Villebon-sur-Yvette, France) with 1 M nitric acid (HNO_3_, POCH, p.a., Gliwice, Poland). The obtained calibration curves showed excellent linearity, with correlation coefficients R^2^ ≥ 0.9995 for all elements. To ensure reliable quantification and control of the total element release, the concentrations in the analyzed solutions were maintained within the calibration range. When necessary, samples were diluted with 1 M nitric acid prior to analysis. The mass of the salt was then calculated from the experimentally determined iron concentration to ensure an equimolar metal-to-metal ratio in the precursor solution.

All metal sulfates were dissolved separately in deionized water produced using a laboratory water purification system (HLP 30, Hydrolab, Straszyn, Poland). Silver sulfate, due to its limited solubility, was dissolved in 500 mL of deionized water to ensure complete dissolution, while iron(III) sulfate hydrate was dissolved in 50 mL of deionized water. After complete dissolution, all prepared solutions were combined under continuous stirring to obtain a homogeneous multimetal sulfate solution targeting an equimolar metal composition. Subsequently, an additional 500 mL of deionized water was added to increase the total reaction volume, thereby lowering the solids content of the suspension after precipitation and improving mixing efficiency.

Co-precipitation was performed using a sodium carbonate solution prepared by dissolving Sodium carbonate (50 g, analytical grade, Sigma-Aldrich, St. Louis, MO, USA) in 300 mL of demineralized water. This amount represented a significant excess relative to the stoichiometric requirement for precipitation of all metal ions, shifting the equilibrium toward the solid phase. The multimetal solution was stirred at 250 rpm for 5 min before the addition of carbonate to ensure homogeneity. The sodium carbonate solution was then introduced, and stirring was continued for 25 min to allow precipitation of the mixed metal carbonates. The concentrations of individual metal ions in solution were determined both before and after the precipitation process using the same AGILENT 4200 MP-AES instrument to evaluate precipitation efficiency. Although carbonate was used as the precipitating agent, it is recognized that not all metals form simple carbonate phases under these conditions. In particular, Fe^3+^ does not form a stable iron(III) carbonate; instead, rapid hydrolysis occurs, leading to the formation of iron(III) hydroxide with simultaneous carbon dioxide evolution, as shown in Equation (1):

Fe_2_(CO_3_)_3_ + 3H_2_O → 2Fe(OH)_3_ + 3CO_2_ (1)

In the case of copper(II), a simple copper carbonate phase is not thermodynamically stable in aqueous systems. Instead, copper carbonate readily transforms into basic copper carbonate (hydroxycarbonate), according to Equation (2):

2CuCO_3_ + H_2_O → Cu_2_(OH)_2_CO_3_ + CO_2_ (2)

It is acknowledged that such transformations occur during the co-precipitation process. However, these differences do not influence the subsequent synthesis pathway, as all obtained carbonates, hydroxycarbonates, and hydroxides undergo thermal decomposition to the corresponding oxides during heating and are subsequently reduced to metallic phases under the applied hydrogen-containing atmosphere. For clarity and consistency throughout the manuscript, the term “carbonates” is therefore used to describe the co-precipitated precursor mixture.

The resulting carbonates were vacuum filtered, rinsed three times with demineralized water to remove residual sodium ions and soluble by-products, and dried in a SML-32/250 laboratory dryer (Zalmed, Skierniewice, Poland) at 90 °C for 24 h. After drying, the carbonates were milled using a commercial model KG200 coffee grinder (De’Longhi, Treviso, Italy) to obtain a homogeneous powder consisting of co-precipitated metal carbonates with equimolar metal composition.

**Figure 1:**
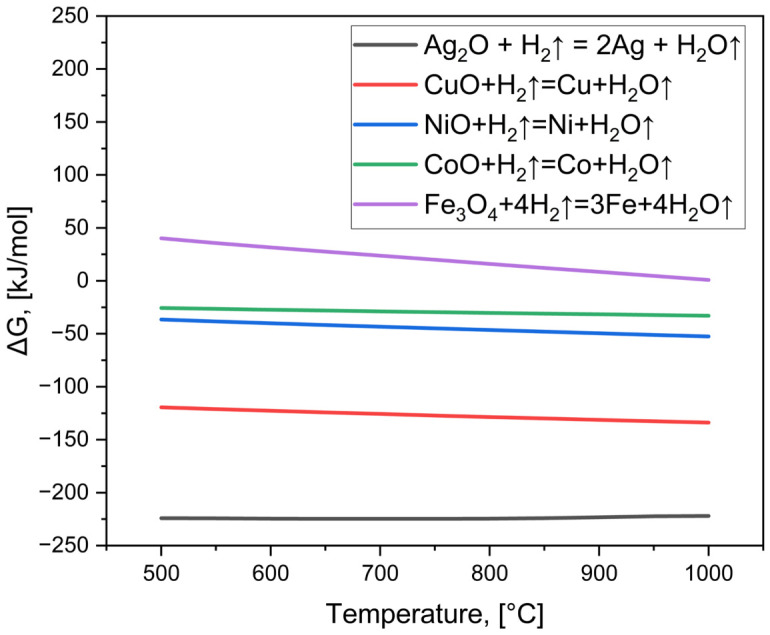
Gibbs free energy changes for reduction in metal oxides present in the discussed system.

The carbonate powder was compacted into cylindrical pellets by uniaxial pressing, with 0.3 g of powder pressed in a 10 mm diameter die under a pressure of 10 MPa. The pressed pellets were subsequently placed in a tube furnace (Czylok, Jastrzębie-Zdrój, Poland) equipped with a quartz tube. Prior to heating, the furnace was purged with a reducing Arcal atmosphere (95% Ar + 5% H_2_, 99.999% purity, Air Liquide, Kraków, Poland) at a gas flow rate of 20 L/h for 0.5 h to ensure complete atmosphere exchange and to guarantee that the reduction process occurred under a fully established reducing environment. The reduction was then conducted at 750 °C with a heating rate of 3 °C/min, and the holding time at the target temperature was varied between 5 and 65 h in 10 h increments. After the reduction treatment, the samples were cooled to room temperature under the same protective atmosphere. To evaluate the feasibility of metal oxide reduction at 750 °C, the Gibbs free energy changes (∆G) for the considered reactions were calculated. The calculations were performed using HSC Chemistry software (version 5.11, Outokumpu research center, Helsinki, Finland), which includes a comprehensive thermodynamic database of various chemical species and enables the determination of ∆G values for specific reactions at a given temperature. It is important to note that the version of the software used does not allow for correction of the partial pressures of individual gases; therefore, the calculations were carried out under the assumption that pH_2_ = pH_2_O. The results of these calculations are presented in Figure 1.

Negative values of ∆G indicate that a given reaction is thermodynamically favorable and can proceed spontaneously under the specified conditions. The obtained results show that for all analyzed metal oxides, except iron oxide, the ∆G values are negative in the calculated temperature range, which suggests that their reduction should occur at 750 °C. In the case of iron oxides, the situation is more complex.

Literature data indicate that the reduction of Fe_2_O_3_ with hydrogen proceeds via two possible pathways depending on the H_2_O/H_2_ ratio [42]. Under conditions of higher humidity, the process follows a three-step mechanism: Fe_2_O_3_ → Fe_3_O_4_ → FeO → Fe (such pathway can also occur during molten salt electrolysis [43]), whereas at low H_2_O/H_2_ ratios it may effectively proceed as a two-step reaction: Fe_2_O_3_ → Fe_3_O_4_ → Fe, due to the rapid reduction of the FeO intermediate. Since Fe_3_O_4_ appears as a common intermediate in both pathways, it was selected as the reference oxide for the thermodynamic calculations. Zieliński et al. demonstrated that the atmosphere in the reactor has a strong influence on the temperature required for reduction to metallic iron. In particular, the H_2_O/H_2_ ratio governs both the mechanism and the temperature range of the process. Under conditions of low humidity and a significant excess of inert gas relative to hydrogen, comparable to those applied in this study, the reduction is considerably facilitated, and the formation of metallic iron may occur at temperatures as low as approximately 592 °C.

In the present study, the reducing gas consisted of 95% Ar and 5% H_2_ with a purity of 99.999%. Assuming a worst-case scenario in which all impurities (0.001%) are attributed entirely to water vapor, the maximum partial pressure of H_2_O can be estimated as pH_2_O ≈ 1 × 10^−5^ atm, while pH_2_ ≈ 0.05 atm, resulting in a ratio pH_2_O/pH_2_ ≈ 2 × 10^−4^. This value is several orders of magnitude lower than the threshold reported in the literature, indicating strongly reducing conditions. Moreover, since a tubular furnace with continuous gas flow was used, it can be assumed that no accumulation of water occurs in the system, and thus the H_2_O/H_2_ ratio remains very low throughout the experiment.

Therefore, it can be reasonably concluded that at 750 °C complete reduction in all metal oxides present in the system will occur.

### 2.2. Characterization of HEA

The surface morphology of the obtained high-entropy alloy (HEA) pellets was examined by scanning electron microscopy (SEM) using a JCM-6000 Plus instrument (JEOL, Tokyo, Japan). Elemental composition and spatial distribution of the constituent elements were analyzed by energy-dispersive X-ray spectroscopy (EDS) using the integrated detector system of the same instrument. SEM-EDS analyses were performed for two surface conditions: the untreated surface and the surface after abrasion (the outer surface layer was manually removed using 400-grit abrasive paper until a uniform, metallically bright surface was obtained), to evaluate elemental distribution and assess compositional homogeneity.

Phase composition was determined by X-ray diffraction (XRD) using a Rigaku MiniFlex II diffractometer (Rigaku Corporation, Tokyo, Japan) equipped with a Cu Kα radiation source (λ = 1.54059 Å). Diffraction patterns were collected in the as-reduced state and after thorough surface abrasion to compare the phase constitution of the surface layer and the underlying material.

The obtained alloy samples were subjected to microhardness measurements using a Shimadzu HMV microhardness tester (Shimadzu Corporation, Kyoto, Japan). The tests were performed under a load of 4.903 N (equivalent to 0.5 kgf), with a dwell time of 10 s applied for each indentation. Prior to testing, the samples were mechanically prepared to ensure a uniform and representative surface condition. The surfaces were first ground using 400-grit silicon carbide abrasive paper to remove surface irregularities and any oxide layer, followed by fine grinding with 1000-grit paper to obtain a smoother finish suitable for reliable indentation measurements. After preparation, the samples were cleaned to remove residual abrasive particles and debris before being placed in the tester. Multiple indentations were performed on each sample to ensure reproducibility and to account for possible microstructural heterogeneity of the alloys.

Surface wettability was evaluated by static contact angle measurements using a high-speed camera (Model 9501, AZ Instrument Corp., Taichung, Taiwan) operated with HiBestViewer software (version 1.0.5.1, AZ Instrument Corp., Taichung, Taiwan). A 1 μL droplet of deionized water was deposited onto the sample surface using the sessile drop method (i.e., analysis of a stationary liquid droplet placed on a flat, solid surface), and the contact angle was determined by contour fitting of the droplet profile using ImageJ Basics software (Version 1.38).

Density measurements were performed using the Archimedes method with an AS 60/220.X2 PLUS analytical balance (Radwag, Radom, Poland) equipped with a density determination kit. Each sample was first weighed in air to determine its mass. Subsequently, the sample was suspended on the lower weighing pan and fully immersed in deionized water of known density, and the apparent mass in the liquid was recorded. All measurements were conducted at a temperature of 21 °C. The density of the solid was calculated according to Archimedes’ principle from the difference between the mass measured in air and the apparent mass measured in the liquid.

SEM/EDS and XRD analyses were performed both on the as-reduced surface and after surface abrasion, allowing direct comparison between the surface layer and the bulk (interior). Microhardness, density, and wettability measurements were carried out after removal of the outer surface layer and therefore characterize the bulk material.

## 3. Results and Discussion

### 3.1. Co-Precipitation of Carbonates

Quantitative elemental analysis of the multimetal system was performed by MP-AES to determine the concentrations of individual metal ions in the aqueous phase before and after the co-precipitation step. The values measured are summarized in Table 1.

The initial concentrations of all five metals were close to the intended equimolar level, confirming accurate preparation of the precursor solution based on stoichiometric calculations and prior analytical determination of iron content. After the co-precipitation step, the concentrations of dissolved metal ions decreased by several orders of magnitude relative to their initial values, indicating extensive transfer of metal species from the liquid phase to the solid precursor. For Co, Ni, and Fe, the concentration decrease values are nearly identical to the initial concentrations, suggesting near-complete removal from solution under the applied conditions. Slightly higher residual concentrations were observed for Ag and Cu, which may be attributed to their comparatively higher solubility in carbonate-containing systems and possible formation of soluble species in the multicomponent environment.

It should be emphasized that the remaining metal concentrations in solution correspond to equilibrium concentrations established in the presence of excess carbonate ions. Due to the applied surplus of CO_3_^2−^, the system was driven toward solid formation. However, complete depletion of dissolved species is thermodynamically unattainable, as solubility equilibria are maintained in the carbonate-rich environment.

Overall, the similarity of the concentration decrease values across all five elements confirms that the co-precipitation strategy enabled the incorporation of each metal at comparable levels, preserving the targeted equimolar character of the precursor mixture.

**Figure 2:**
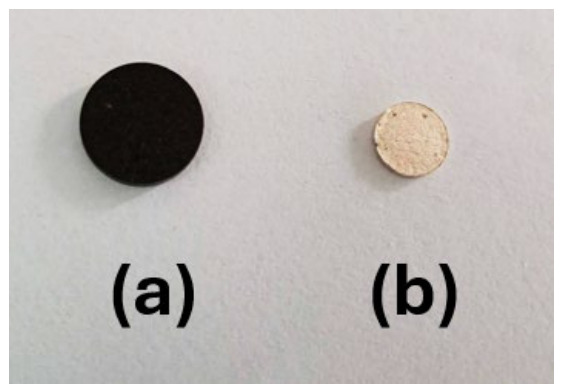
Cylindrical pellet before (**a**) and after (**b**) the reduction process (reduction time = 65 h).

Following co-precipitation, the obtained carbonate precursors were compacted into cylindrical pellets and subsequently subjected to a reduction process carried out under varying holding times, as specified in Section 2. Figure 2 shows a sample before and after the reduction process, illustrated by a representative pellet obtained at a holding time of 65 h.

### 3.2. SEM and EDS Analysis

**Figure 3:**
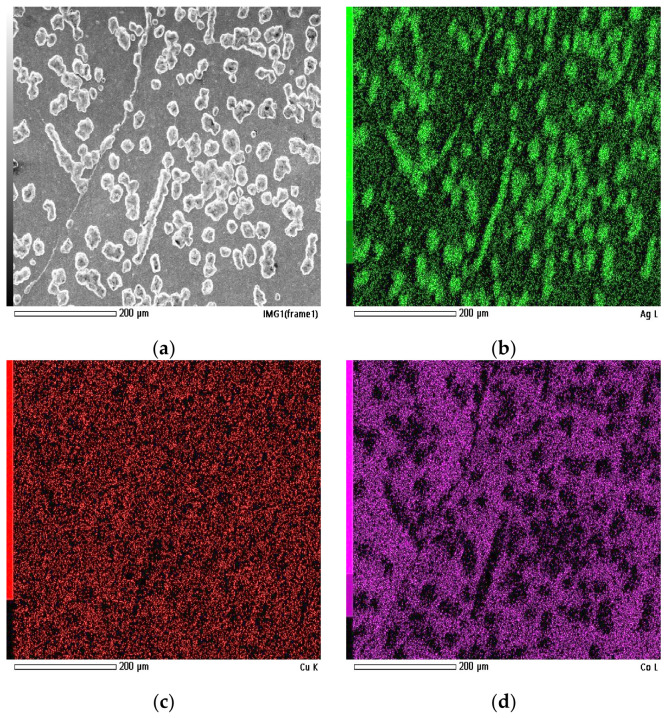

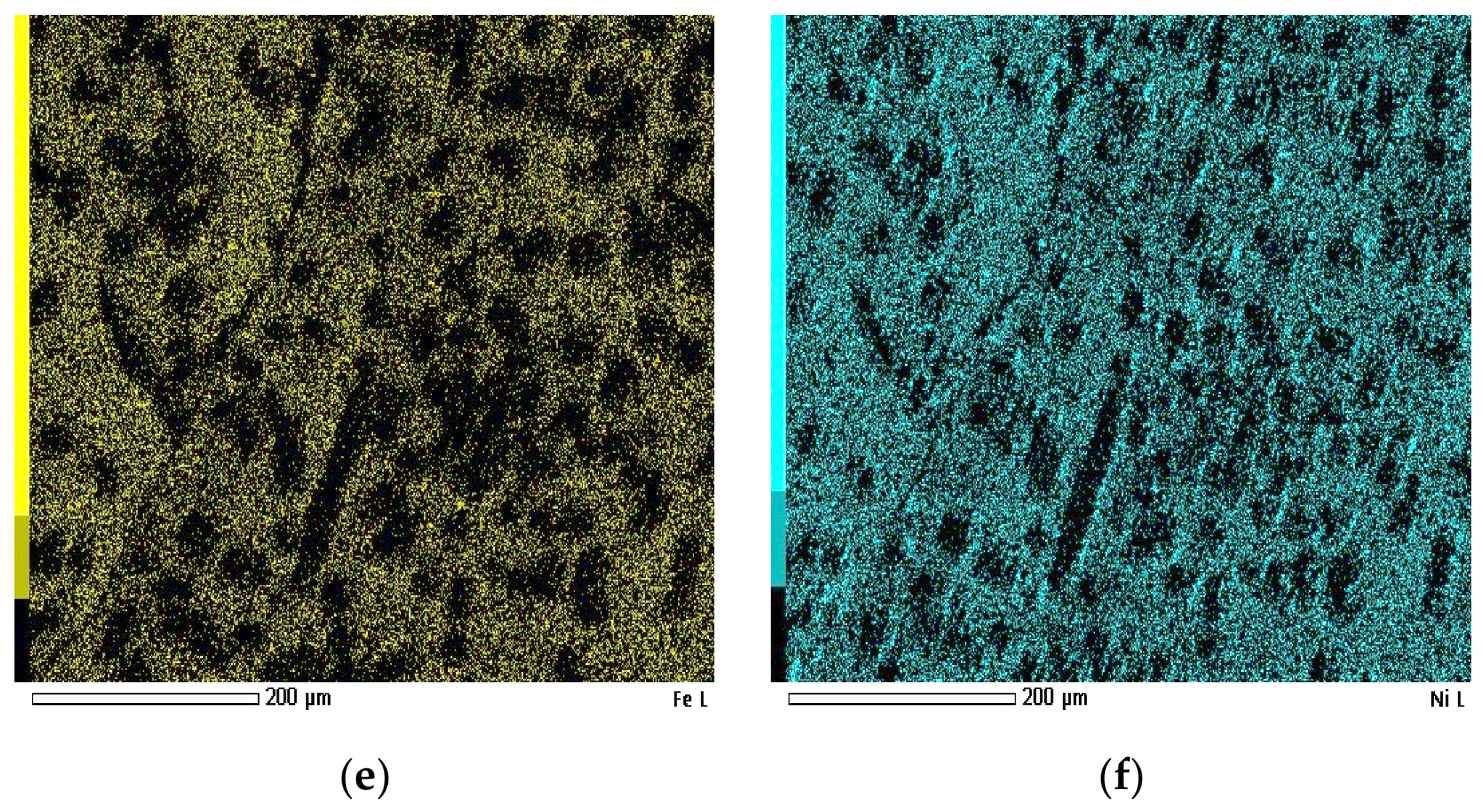
EDS analysis of the as-reduced HEA pellet surface: (**a**) SEM image of the analyzed area; elemental distribution maps of (**b**) Ag, (**c**) Cu, (**d**) Co, (**e**) Fe, and (**f**) Ni.

SEM and EDS analyses were performed on the HEA pellet synthesized with a holding time of 65 h to evaluate its surface characteristics and elemental distribution. This sample was selected as representative of the longest reduction duration. The analysis was conducted for two surface conditions: in the as-reduced state and after thorough surface abrasion, allowing comparison between the outer surface layer and the underlying material. The corresponding EDS elemental maps for the as-reduced condition are presented in Figure 3. Quantitative results obtained from the mapped area are summarized in Table 2.

SEM image reveals a heterogeneous surface with clearly distinguishable precipitate-like features distributed across the surface. These features appear as bright regions in backscattered electron contrast, indicating compositional differences relative to the surrounding matrix. The morphology suggests surface segregation of Ag, resulting in Ag-enriched domains concentrated at the outer surface layer.

The EDS maps confirm the presence of Ag-enriched surface domains, consistent with the contrast variations observed in the SEM image. The Ag signal is predominantly localized within the bright regions, while Cu is also clearly present within these segregated areas. In contrast, Co, Fe, and Ni exhibit a more dispersed distribution across the analyzed surface, indicating their predominant presence within the matrix phase.

Quantitative EDS analysis (Table 2) reveals the following atomic fractions: Fe—25.50 at.%; Co—20.95 at.%; Ni—25.75 at.%; Cu—15.12 at.%; and Ag—12.68 at.%. The composition deviates from the targeted equimolar ratio, particularly due to the reduced atomic fraction of Ag and Cu relative to Fe, Co, and Ni. This deviation is consistent with the observed surface segregation behavior, where Ag-rich domains are locally concentrated rather than uniformly distributed across the surface.

The comparatively lower average Ag content detected by EDS, despite visible Ag-rich segregations, suggests that the analyzed area includes both Ag-enriched regions and matrix regions with lower Ag concentration. As a result, the measured atomic percentage represents an averaged value over heterogeneous surface domains. The presence of Cu both within Ag-rich features and in the surrounding matrix explains its intermediate atomic fraction. Observed Ag-enriched regions may be tentatively related to compositional heterogeneity reported in Ag-Cu systems, although this interpretation remains speculative and is not directly supported by experimental evidence in the present study. At equilibrium, the eutectic temperature is around 779 °C [44]. At this temperature, the alloy solidified with an Ag-rich composition. A. Munitz and others observed that addition of silver to AlCoCrCuNi or AlCoCrCuFeN HEAs leads to liquid phase separation into two melts—an AlCoCr(Fe) rich liquid and Ag and Cu rich second liquid [45]. Differential thermal analysis (DTA) results show that the eutectic reaction occurred at 780 °C. At the same time, U.S. Hsu and others observed a globule-like phase formed from Ag liquid droplet trapped in a layer of Al, Co, Cr, and Ni, with a small amount of Cu. Depending on the casting methods and cooling rates, different microstructures can be observed [46]. With the higher cooling rate, the eutectic point moves towards the higher Cu concentration. It is worth noting that eutectic high-entropy alloys are gaining increasing interest due to their enhanced mechanical properties compared with standard HEAs [47]. In the context of the present study, however, the interpretation in terms of eutectic-related behavior and phase separation should be regarded as a qualitative hypothesis rather than a confirmed mechanism.

**Figure 4:**
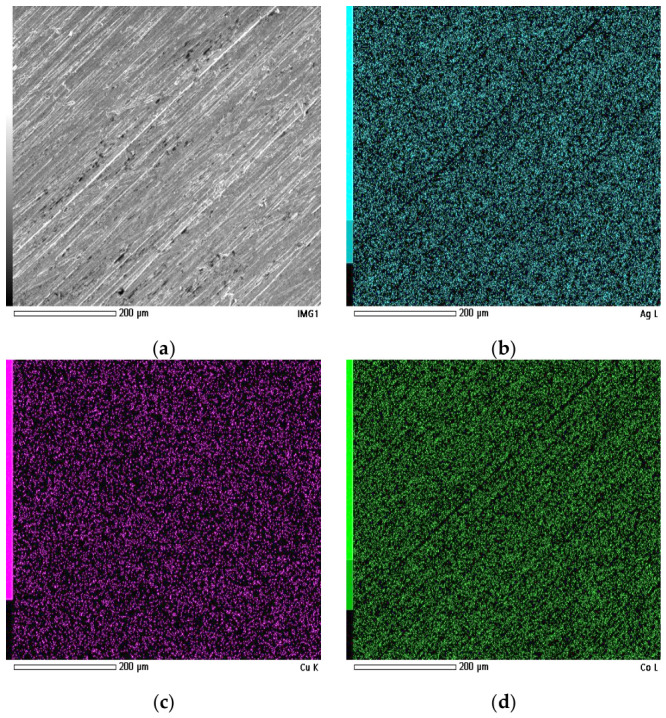

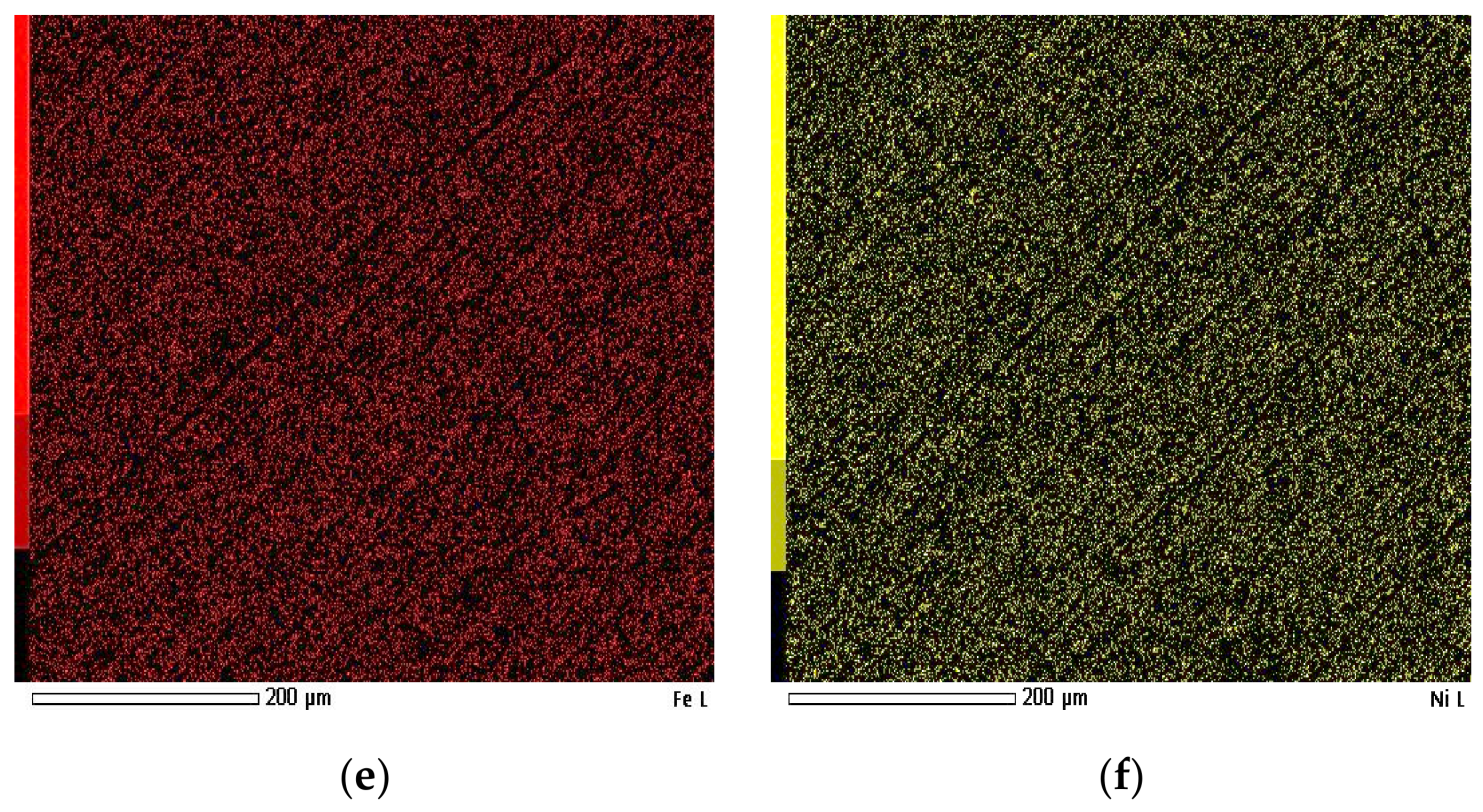
EDS analysis of the HEA pellet surface after thorough abrasion: (**a**) SEM image of the analyzed area; elemental distribution maps of (**b**) Ag, (**c**) Cu, (**d**) Co, (**e**) Fe, and (**f**) Ni.

SEM image recorded after thorough surface abrasion and corresponding EDS elemental maps are shown in Figure 4, while the quantitative atomic composition obtained from the mapped area is summarized in Table 3.

In contrast to the as-reduced condition, the abraded surface exhibits a considerably more uniform morphology. No distinct surface segregation features are observed, and the surface appears relatively uniform at the microscale, apart from linear features associated with mechanical abrasion marks.

The elemental distribution across the analyzed surface is markedly more uniform compared to the as-reduced condition. No localized Ag-rich domains are detected, and all five elements are dispersed relatively evenly over the investigated area.

Quantitative analysis reveals the following atomic fractions: Fe—25.55 at.%, Co—22.52 at.%, Ni—29.18 at.%, Cu—11.71 at.%, and Ag—11.04 at.%. Compared to the as-reduced surface, the Ag and Cu contents decrease slightly, while Ni and Co exhibit a moderate increase. This shift in average composition supports the conclusion that the previously observed Ag surface segregation was primarily confined to the outer surface layer and was removed during abrasion, revealing a more uniform, although not fully chemically homogeneous composition.

**Figure 5:**
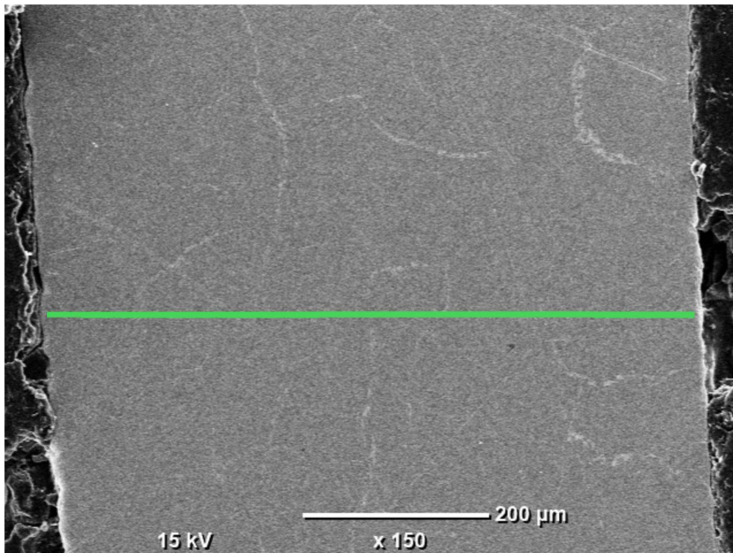
SEM image of the HEA pellet cross-section with marked EDS line analysis locations.

**Figure 6:**
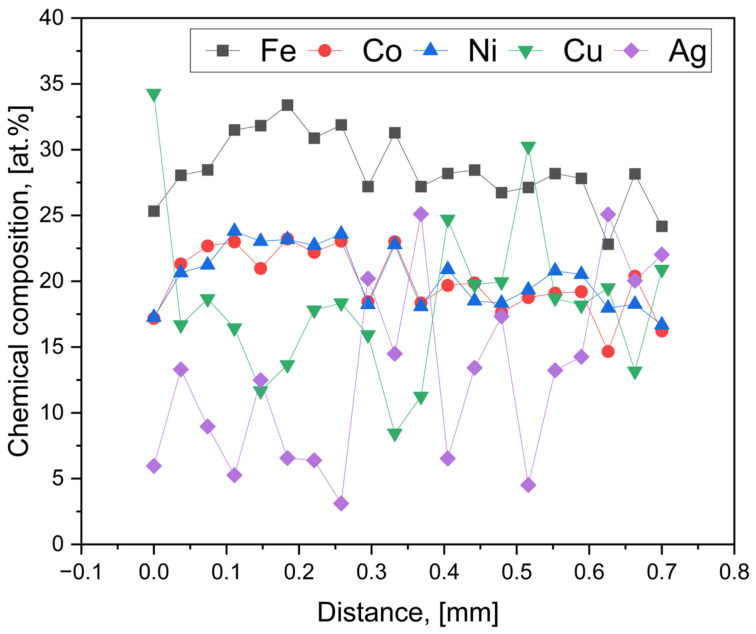
Chemical composition along the green line marked in Figure 5.

To further clarify the compositional differences observed between the as-reduced and abraded surfaces, an additional EDS investigation was performed on the cross-section of the HEA pellet. In this case, a linear point analysis consisting of 20 measurement locations was carried out across the sample cross-section, enabling comparison of elemental composition between the near-surface region and the interior of the pellet. While this approach does not allow for direct visualization of potential segregation phenomena, it provides quantitative insight into compositional variations along the analyzed line. The SEM image with the marked measurement points is presented in Figure 5, and the corresponding quantitative results are summarized in Figure 6.

The cross-sectional EDS point analysis reveals noticeable compositional fluctuations along the analyzed line, confirming that the HEA pellet is not fully homogeneous across its thickness. The average composition (Fe 28.43 at.%, Co 19.94 at.%, Ni 20.29 at.%, Cu 18.41 at.%, Ag 12.92 at.%) remains broadly consistent with the surface measurements; however, the relatively high standard deviations, particularly for Cu (±6.08 at.%) and Ag (±6.96 at.%), indicate significant local variability. In contrast, Fe, Co, and Ni exhibit remarkably stable compositions across all analyzed points, with low standard deviations (±2.71, ±2.48, and ±2.31 at.%, respectively), indicating that these elements form a chemically uniform and continuous matrix throughout the cross-section. Their distributions show only minor fluctuations and no localized enrichment or depletion, highlighting their dominant role in stabilizing the bulk phase of the alloy.

On the other hand, distinct enrichment of Ag is observed at several points, where its content exceeds 20 at.%, while other regions exhibit significantly lower Ag concentrations. A similar trend is observed for Cu, with localized peaks reaching over 30 at.%, further confirming the presence of compositional heterogeneity within the material. The absence of a clear monotonic trend from one edge of the sample to the other suggests that these variations are not governed by a simple surface-to-core gradient but rather reflect localized microsegregation within the bulk. The underlying mechanisms responsible for this behavior are not directly identified in the present study and remain a subject of interpretation. These findings are consistent with the earlier surface observations, reinforcing the conclusion that Ag and Cu tend to segregate, whereas the Fe–Co–Ni-rich matrix remains highly homogeneous across the entire pellet.

The average composition determined from the cross-sectional EDS analysis deviates from the ideal equimolar ratio, with Fe, Co, and Ni present at higher atomic fractions compared to Cu and Ag. This deviation suggests that the intended equimolar distribution of elements was not fully achieved under the applied processing conditions. Importantly, it should be noted that achieving a perfectly equimolar composition is not a strict requirement for classification as a high entropy alloy. The defining criterion is the configurational entropy of mixing (ΔS_mix_). For the present composition, the mixing entropy can be estimated using the following equation:

(3) $$∆S_{mix}=-R\sum x_{i}\ln x_{i}$$

Substituting the experimentally determined average atomic fractions (x_i_) yields a value of ΔS_mix_ ≈ 1.578R. This exceeds the commonly accepted threshold of 1.5R for high entropy alloys, confirming that the investigated system can be classified as a high entropy alloy despite deviations from equimolarity. However, compositional fluctuations observed across the cross-section indicate that the alloy is not fully chemically homogeneous.

### 3.3. XRD Analysis

**Figure 7:**
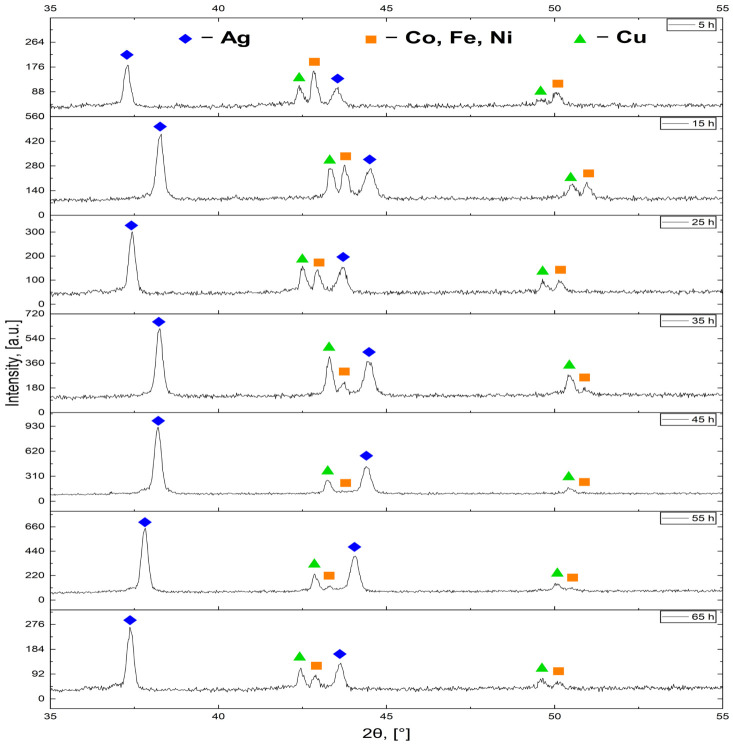
XRD patterns of HEA pellets synthesized with reduction times ranging from 5 to 65 h, recorded before surface abrasion.

The phase evolution of the HEA pellets as a function of reduction time was investigated by XRD. Diffraction patterns recorded for samples before surface abrasion are presented in Figure 7, while selected peak parameters are presented in Table 4.

Before surface abrasion, all samples exhibit sharp and well-defined diffraction maxima, indicating high crystallinity and relatively low lattice distortion within the diffracting phases. The most characteristic feature of the non-abraded condition is observed in the 42–44° 2θ interval, where three narrow and clearly separated reflections are consistently resolved at all holding times. This angular range corresponds to reflections associated with FCC metals present in the alloy, particularly Cu(111) and Ag(200), while the central peak arises from the overlapping contributions of Co(111), Ni(111), and Fe(111), indicating the presence of segregated crystalline regions enriched in specific elements within the surface layer. Although their exact angular positions vary slightly between samples, as shown in Table 5, this triplet configuration persists throughout the investigated time range. At 45 h, the central reflection becomes noticeably broadened and visually less distinct; however, three components remain numerically identifiable. Thus, within the 42–44° range, peak multiplicity is maintained before surface modification, with changes occurring primarily in peak sharpness rather than in the number of resolved contributions.

**Figure 8:**
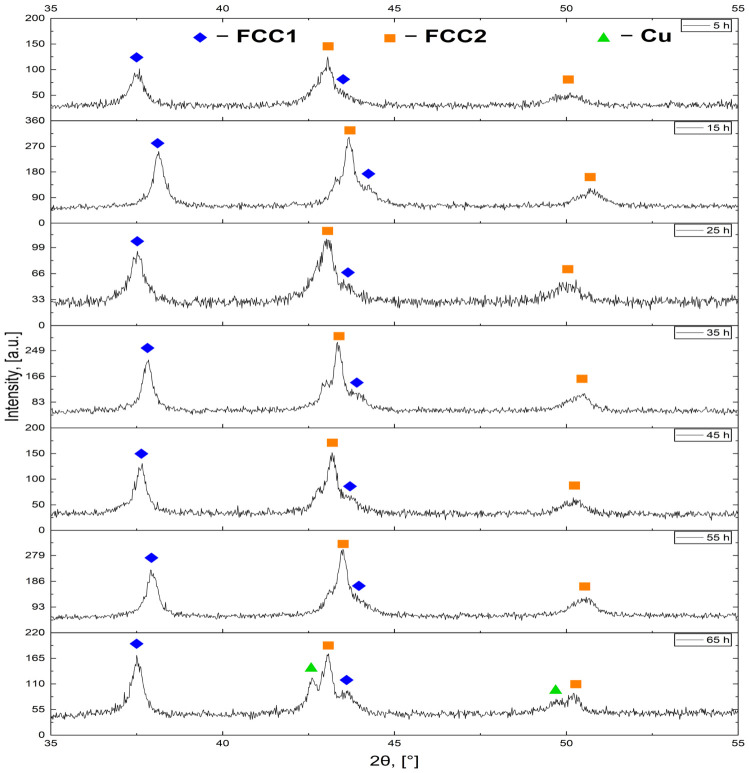
XRD patterns of HEA pellets synthesized with holding times ranging from 5 to 65 h, recorded after surface abrasion.

In the lower-angle region near 37–38°, a single dominant reflection is present at each holding time before abrasion. This peak corresponds to the (111) reflection characteristic of FCC Ag, suggesting the presence of Ag-rich domains within the outer surface layer. Its position fluctuates within a limited angular window and does not exhibit a systematic monotonic shift with increasing holding time. Comparable small positional variations are observed in a 49–51° range; these reflections can be associated with Cu(200) and Co,Ni,Fe(200) planes. Overall, the pre-abrasion patterns are characterized by sharp peaks and persistent multiplicity in the 42–44° region. The diffraction patterns obtained after thorough surface abrasion are presented in Figure 8, while selected peak parameters are presented in Table 5.

In contrast to the non-abraded condition, the 42–44° 2θ interval is dominated at all holding times by a single broad and intense reflection, now identified as FCC2(111). In the pre-abrasion diffractograms, the reflections were narrow and well-separated, allowing unambiguous assignment to individual metallic phases—Cu, Ag, and a Co/Ni/Fe phase—with limited peak overlap and relatively small FWHM values consistent with a relatively low degree of alloying. Under these conditions, retaining the individual metal designations was justified. Following abrasion, however, the three distinct reflections within the 42–44° window collapse into a single dominant peak, and the general broadening of reflections across all principal angular regions suggests an increased degree of alloying. This justifies the transition to a phase-based nomenclature—FCC1 and FCC2—reflecting the fact that the observed reflections can no longer be reliably attributed to individual elemental metals, but rather to alloyed FCC phases of distinct compositions.

Nevertheless, the FCC2(111) peak is not perfectly symmetric. A weak shoulder on its high-angle flank is consistently visible and is assigned to FCC1(200), corresponding to an Ag-rich FCC phase. This two-phase character is likely associated with the positive mixing enthalpy between Ag and the remaining constituent metals—Cu, Co, Ni, and Fe—combined with the atomic radius mismatch, both of which limit the incorporation of Ag into the common FCC lattice, causing it to appear as a crystallographically distinct phase in the diffraction profile [48]. Additionally, a small feature on the low-angle side of the FCC2(111) peak may be attributed to residual Cu(111), though this contribution remains weak and largely unresolved across most holding times. Only at 65 h does a clearly visible Cu reflection emerge—specifically the Cu(200) reflection near 50.4° 2θ—suggesting that at extended reduction times, a fraction of Cu retains sufficient crystallographic coherence to produce a discernible diffraction signal.

The reflections in the post-abrasion condition are generally broader than those observed before abrasion, not only within the 42–44° interval but also in the FCC1(111) region near 37–38° and in the 49–51° window. The broadening of both FCC1 and FCC2 reflections across all holding times may be associated with alloying in a multicomponent system; however, peak broadening can also arise from other factors, such as reduced crystallite size or microstrain. Across both conditions, peak positions fluctuate within relatively narrow angular windows and do not follow a systematic linear trend with holding time. The most pronounced structural change between the two conditions is therefore not a continuous shift in peak position, but rather the collapse of peak multiplicity and the accompanying increase in peak breadth after surface abrasion.

Based strictly on the observed 2θ behavior, the non-abraded samples exhibit persistent peak multiplicity in the 42–44° interval, whereas the abraded samples display a single broadened reflection in the same range. This evolution from multiple sharp reflections to fewer broadened peaks supports the interpretation of increased chemical mixing and structural evolution within the bulk, although this conclusion remains qualitative.

Overall, the observed changes in peak shape, position, and multiplicity provide qualitative evidence of enhanced alloying within the bulk compared to the surface and reflect the structural differences between the outer layer and the interior of the material, and should be interpreted as qualitative, with more detailed phase identification requiring advanced quantitative analysis beyond the scope of this work.

### 3.4. Microhardness Measurements

**Figure 9:**
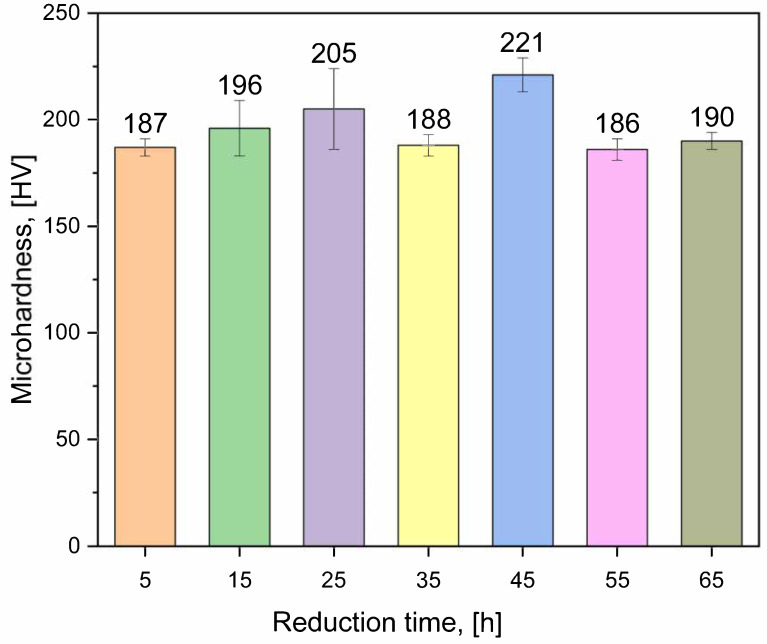
Influence of time of reduction on measured microhardness.

Possible residuals of metal carbonates could influence the mechanical properties of alloys; therefore, the microhardness of samples with reduction times ranging from 5 to 65 h was measured six times for each. Prior to the measurements, all samples were subjected to surface abrasion in order to ensure reliable and consistent microhardness results. Results are shown in Figure 9.

There is no clear influence of the reduction time on the alloys’ microhardness. The obtained results suggest that the reduction time could be shortened to 5 h. All HEAs are moderately hard; therefore, they can be formed or easily machined if required for future applications. The literature review shows that there are no microhardness values measured for the high-entropy alloys with a similar composition. A 4.5 mm thick Al-Co-Cr-Fe-Mn-Ni HEA coating shows hardness of 294 ± 53 HV [49]. For as-cast Cu-Ag-Fe alloys, the maximum hardness value (around 150 HV2) was observed at 2.4 vol% Fe [50]. It relates to more Fe precipitates observed as they lead to a higher hardening effect than precipitates of Ag. AlCoCrCuNi-based equimolar high-entropy alloys prepared by the arc melting and casting method show BCC and FCC phases and a typical cast structure with dendrites [51]. Due to the positive mixing enthalpies between Cu and Co, Cr and Ni, copper segregation to the interdendrite region appeared. Hardness of this alloy was 419 ± 6 HV. The addition of Fe to this alloy again caused the segregation of Cu to the interdendrite region, as mixing enthalpies between Fe and Al, Co, Cr, and Ni are negative, and between Fe and Cu, it is positive. The hardness of this alloy was 416 ± 19 HV. When Ag is added to the AlCoCrCuNi alloy, two separate layers of silver and gold colors were observed. The silver layer was composed of Al, Co, Cr, and Ni, with a smaller amount of Cu, and the gold layer consisted of Ag with some Cu. They showed significantly different hardness values, 104 ± 3 HV and 451 ± 9 HV for gold and silver layers, respectively. Copper segregated to the golden layer was the reason for the highest hardness of the silver layer. Similar measured values of microhardness in this work suggest a relatively uniform mechanical response, although compositional heterogeneity is still present within the material. It was found that for nearly all HEAs the hardness equals to three times the magnitude of strength [52].

### 3.5. Density Measurements

**Figure 10:**
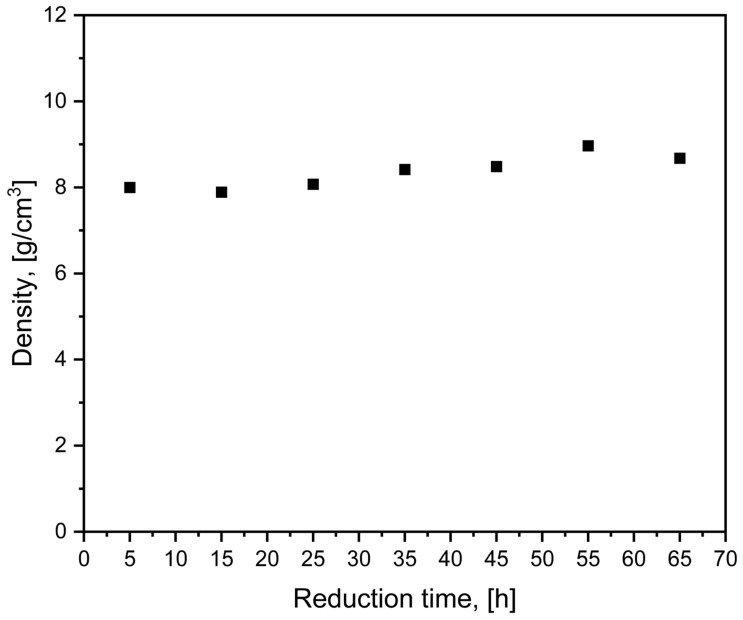
Density of HEA pellets as a function of reduction time.

Density measurements were carried out to evaluate the influence of reduction time on the densification of the synthesized HEA pellets. Although phase formation and alloying were confirmed by the structural and microstructural analyses discussed earlier, the reduction duration can still affect the compactness of the pellets through changes in residual porosity and particle bonding during processing. Therefore, density measurements provide complementary information on the physical consolidation of the pellets obtained under different reduction conditions. The measured density values of the investigated samples (after surface abrasion) are presented in Figure 10.

The evolution of the relative density of the AgCuCoNiFe pellets, as shown in Figure 10, reveals a progressive densification trend up to a reduction time of 55 h, followed by an unexpected decline at the 65 h mark. This behavior stands in contrast to previous findings regarding pure silver systems synthesized via the carbonate-reduction route [53], where silver clays exhibited stable densification and high final conductivity. In the present multicomponent system, the initial stages of thermal treatment are associated with significant volume shrinkage resulting from the decomposition of the carbonate precursor and subsequent sintering of the newly formed metallic phases. As the reduction time increases, enhanced particle bonding and diffusion-driven consolidation contribute to the observed increase in density.

However, the decrease in density observed for the longest reduction time (65 h), despite the absence of visible macroscopic swelling or deformation, suggests the onset of additional microstructural processes that counteract further densification. One possible explanation may be related to differences in intrinsic diffusion rates among the constituent elements (Ag, Cu, Co, Ni, Fe), which can lead to vacancy imbalance during interdiffusion. Such diffusion asymmetry may promote the formation of submicron voids or vacancy clusters within the material. Although this type of behavior is sometimes associated with the Kirkendall effect, it should be emphasized that no direct experimental evidence is available in the present study to confirm this mechanism. Therefore, this interpretation should be regarded as a speculative explanation and is not directly confirmed by the experimental data obtained in this study.

### 3.6. Wettability Measurements

**Figure 11:**
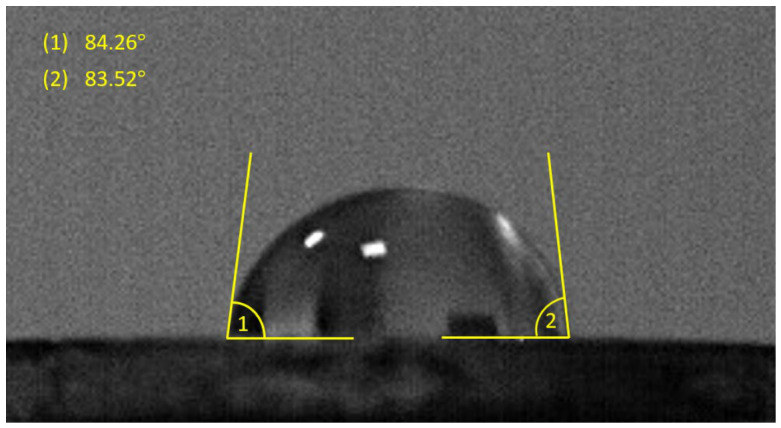
Sessile drop contact angle measurement methodology.

The wettability of the obtained HEA surfaces was evaluated after removal of the outer surface layer in order to determine how the reduction time influences the interaction between the alloy surface and water. Changes in contact angle values may reflect variations in the surface state of the material, including differences in near-surface composition, oxidation behavior, or structural heterogeneity resulting from the thermal treatment. An example of the recorded droplet profile used for contact angle determination is presented in Figure 11.

**Figure 12:**
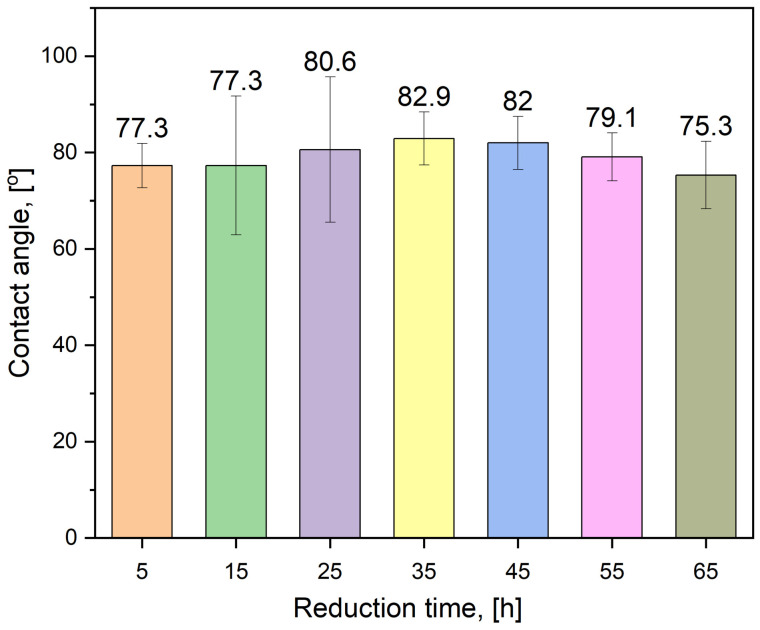
Values of contact angles.

The contact angle values obtained for all investigated samples are summarized in Figure 12. Each value represents the average obtained from multiple measurements performed on samples reduced for different holding times. The comparison of these values allows evaluation of possible changes in surface wettability associated with variations in the thermal treatment duration.

The contact angle of the HEA samples exhibits a dependence on the reduction time, while the observed trend is non-monotonic. The average contact angle values fall within the range of ~75.3–82.9°, corresponding to moderate hydrophilicity to borderline hydrophobicity.

The most stable result was obtained for the sample reduced for 5 h (77.28° ± 4.56°), indicating a relatively uniform surface state at the macroscopic scale already at short reduction times. For the samples reduced for 15 h and 25 h, the average contact angle values are 77.32° ± 14.40° and 80.58° ± 15.10°, respectively; however, these values are accompanied by a very large scatter (including distinct outliers), suggesting pronounced surface heterogeneity within this time window. This behavior may be associated with non-uniform degrees of reduction/oxidation or with local microstructural and topographical variations.

In the range of 35–45 h, a stabilization at the highest average wettability level is observed, with values of 82.92° ± 5.46° (35 h) and 82.01° ± 5.51° (45 h), accompanied by low and comparable standard deviations. This trend is consistent with the progressive homogenization of the surface state, likely resulting from continued reduction combined with interdiffusion and compositional leveling within the near-surface layer.

For longer reduction times, a decrease in the average contact angle is observed: 79.11° ± 5.00° (55 h) and 75.26° ± 7.02° (65 h). The simultaneous increase in data scatter for the 65 h sample indicates that prolonged thermal treatment may induce secondary surface-related effects, such as local elemental segregation, reconstruction of the oxide layer upon air exposure, or changes in surface roughness, leading to increased wettability heterogeneity.

In accordance with the approach proposed by Skibińska et al. [54], variations in the contact angle should be interpreted primarily as a consequence of surface-energy modifications arising from changes in near-surface chemistry—particularly the relative contributions of metallic and oxidized species—rather than as a direct function of surface morphology or heat-treatment duration alone. In the cited work, significantly lower contact angle values (≈30–60°) were reported for nanostructured Co-Ni coatings in the fresh and controlled-oxidation states, which the authors attributed to the presence of active transition-metal oxides and a highly developed conical surface morphology. In contrast, the higher contact angle values observed for the present HEA samples (≈75–83°) can be ascribed to the different synthesis route (co-precipitation followed by reduction), leading to a more homogeneous, alloyed surface layer with a limited contribution of stable oxides and a less pronounced topographical development. Consequently, the differences in contact angle values are consistent with distinct chemical and structural surface states, while preserving a comparable non-monotonic wettability response to surface modification. Literature review shows that, depending on their thickness, CoCrFeMnNi high-entropy alloy films exhibit contact angle values of up to 96° [55]. There are a few approaches to obtain hydrophobic HEAs, e.g., precise control of their composition by radio frequency magnetron sputtering, allowing to obtain water contact angle of 129 ± 0.5° for AlCoCrCu0.5FeNi HEA [56]. Another method is alloys’ modification, e.g., with electrochemical dealloying of AlCoCrFeNi HEA [57] or surface modification of ZnFeCoNiMn high-entropy alloy coating with tetradecanoic acid [58]. These approaches allow for contact angles up to 127° and 154°, respectively. Therefore, the hydrophilic, nearly hydrophobic properties of HEAs, presented in this work, could be expected.

## 4. Conclusions

A compositionally complex two-phase AgCuCoNiFe high-entropy alloy was successfully synthesized using a precipitation-reduction route involving co-precipitation of metal carbonates followed by controlled thermal treatment. The resulting composition is not equimolar, which can be attributed to surface segregation effects and the persistence of equilibrium metal concentrations in solution during the precipitation process. Nevertheless, the key criterion for classification as a high entropy alloy—namely, a configurational entropy of mixing exceeding 1.5R—was achieved.

Structural analysis revealed that the outer surface layer exhibited compositional heterogeneity arising from Ag surface segregation, with Ag-enriched domains concentrated at the surface. After abrasion, the bulk material displayed a more uniform elemental distribution at the surface, although cross-sectional analysis confirmed that full chemical homogeneity was not achieved. Diffraction patterns indicated a clear structural distinction between the surface and the interior, with the bulk exhibiting a two-phase character with broadened reflections consistent with increased lattice distortion and chemical disorder typical of alloying in multicomponent systems.

Variation in reduction time did not produce significant changes in microhardness, indicating that prolonged thermal treatment beyond the shortest investigated duration is not necessary to achieve stable mechanical properties. Density measurements revealed a general increase in pellet density with increasing reduction time, suggesting gradual densification of the material during thermal treatment, although minor deviations from a strictly monotonic trend were observed.

Wettability measurements showed moderate hydrophilicity with a non-monotonic dependence on reduction time, reflecting surface-energy variations associated with near-surface compositional and structural modifications.

Overall, the results confirm that the precipitation-reduction strategy can be effectively extended to the synthesis of compositionally complex two-phase high-entropy alloys, providing a viable alternative to conventional high-temperature processing routes. However, the obtained results do not indicate any particularly distinctive properties of the investigated alloy that would currently justify its direct industrial application. Nevertheless, the applied synthesis approach has been clearly demonstrated to be effective and may be successfully utilized for the preparation of high-entropy alloys. At the same time, the observed compositional heterogeneity of the synthesized material highlights the need for further optimization of the process parameters to achieve a more uniform alloy structure and fully exploit the potential of this method.

## Figures and Tables

**Table 1:** Concentrations of metal ions in solution before and after co-precipitation.

Metal	Initial Concentration [mol/L]	Equilibrium Concentration [mol/L]	Precipitation Yield [%]	Atomic Fraction in Precipitate [%]
Ag	0.03973	1.76 × 10^−4^	99.56	20.02
Cu	0.04045	3.43 × 10^−4^	99.15	20.30
Co	0.04014	5.09 × 10^−6^	99.99	20.31
Ni	0.03895	2.73 × 10^−6^	99.99	19.71
Fe	0.03884	1.02 × 10^−5^	99.97	19.65

**Table 2:** Atomic composition of the as-reduced HEA surface determined by EDS mapping.

Metal	Atomic Fraction [%]
Ag	12.68
Cu	15.12
Co	20.95
Ni	25.75
Fe	25.50

**Table 3:** Atomic composition of the HEA surface after thorough abrasion determined by EDS mapping.

Metal	Atomic Fraction [%]
Ag	11.04
Cu	11.71
Co	22.52
Ni	29.18
Fe	25.55

**Table 4:** XRD peak parameters for Ag (111) and Co,Ni,Fe (111).

Reduction Time, [h]	Ag(111)		Co,Ni,Fe(111)	
	2θ, [°]	FWHM	2θ, [°]	FWHM
5	37.258	0.179	42.827	0.139
15	38.241	0.202	43.730	0.158
25	37.405	0.186	42.928	0.145
35	38.216	0.188	43.702	0.185
45	38.177	0.189	43.685	1.324
55	37.776	0.182	43.297	0.157
65	37.361	0.186	42.877	0.154

**Table 5:** XRD peak parameters for FCC1(111) and FCC2(111).

Reduction Time, [h]	FCC1(111)		FCC2(111)	
	2θ, [°]	FWHM	2θ, [°]	FWHM
5	37.521	0.485	42.998	0.675
15	38.109	0.326	43.214	0.269
25	37.437	0.473	42.978	0.661
35	37.802	0.282	42.984	0.668
45	37.612	0.332	43.127	0.386
55	37.917	0.352	43.432	0.387
65	37.497	0.371	43.075	0.276

## Data Availability

The original contributions presented in this study are included in the article. Further inquiries can be directed to the corresponding author.
